# Supplementary material for: Climate-related factors cause changes in the diversity of fish and invertebrates in subtropical coast of the Gulf of Mexico
Source: Commun Biol. 2019 Nov 1;2:403. doi: 10.1038/s42003-019-0650-9 (PMC6825143; doi:10.1038/s42003-019-0650-9)
Supplement: Supplementary file 1 — Supplementary Information [file 42003_2019_650_MOESM1_ESM.pdf]

Supplementary Figures

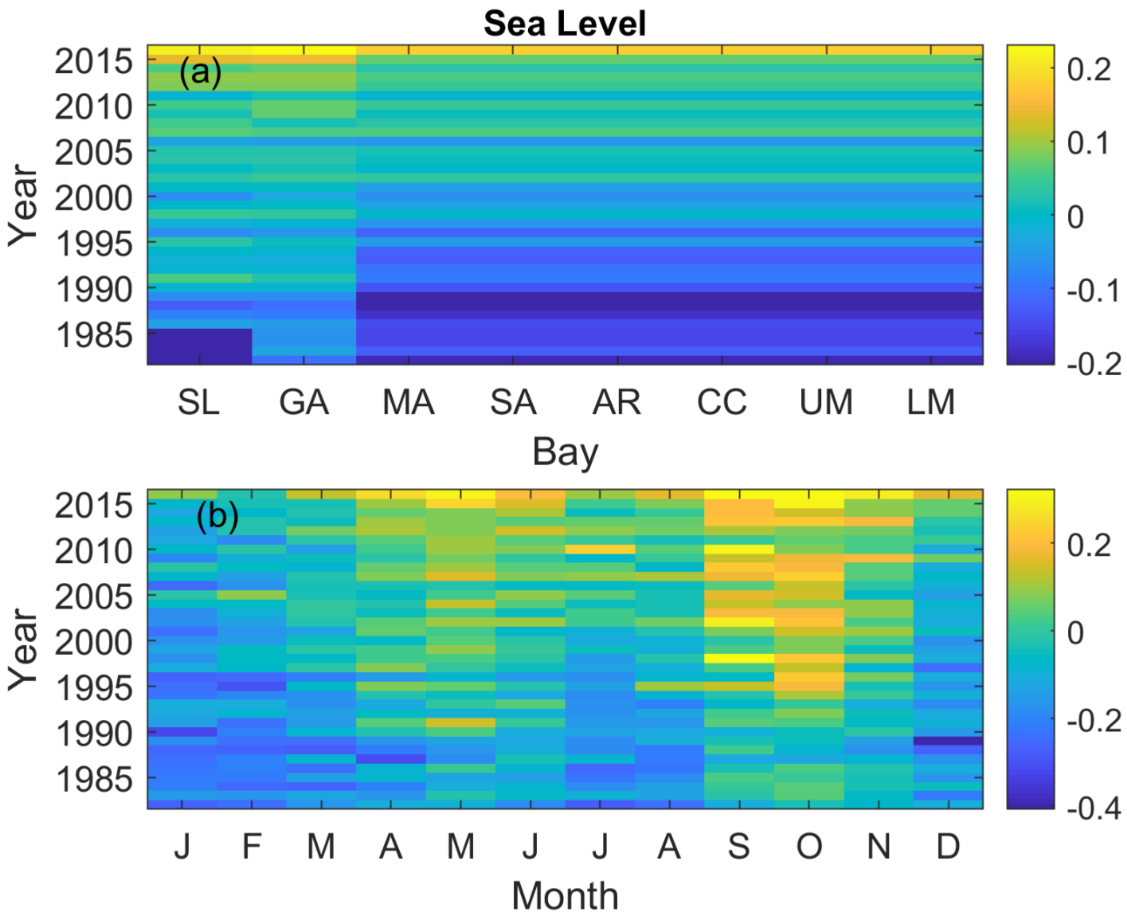

Supplementary Figure 1. The monthly mean sea level (m) used in the analysis. The locations of the data are shown in Supplementary Table 1. (a) Averaged over year to show spatial variability, (b) Averaged among locations to show monthly variability. SL: Sabine Lake, GA: Galveston Bay, MA, Matagorda Bay, SA: San Antonio Bay, AR: Aransas Bay, CC: Corpus Christi Bay, UM: Upper Laguna Madre, and LM: Lower Laguna Madre. J-D denote months of a year.

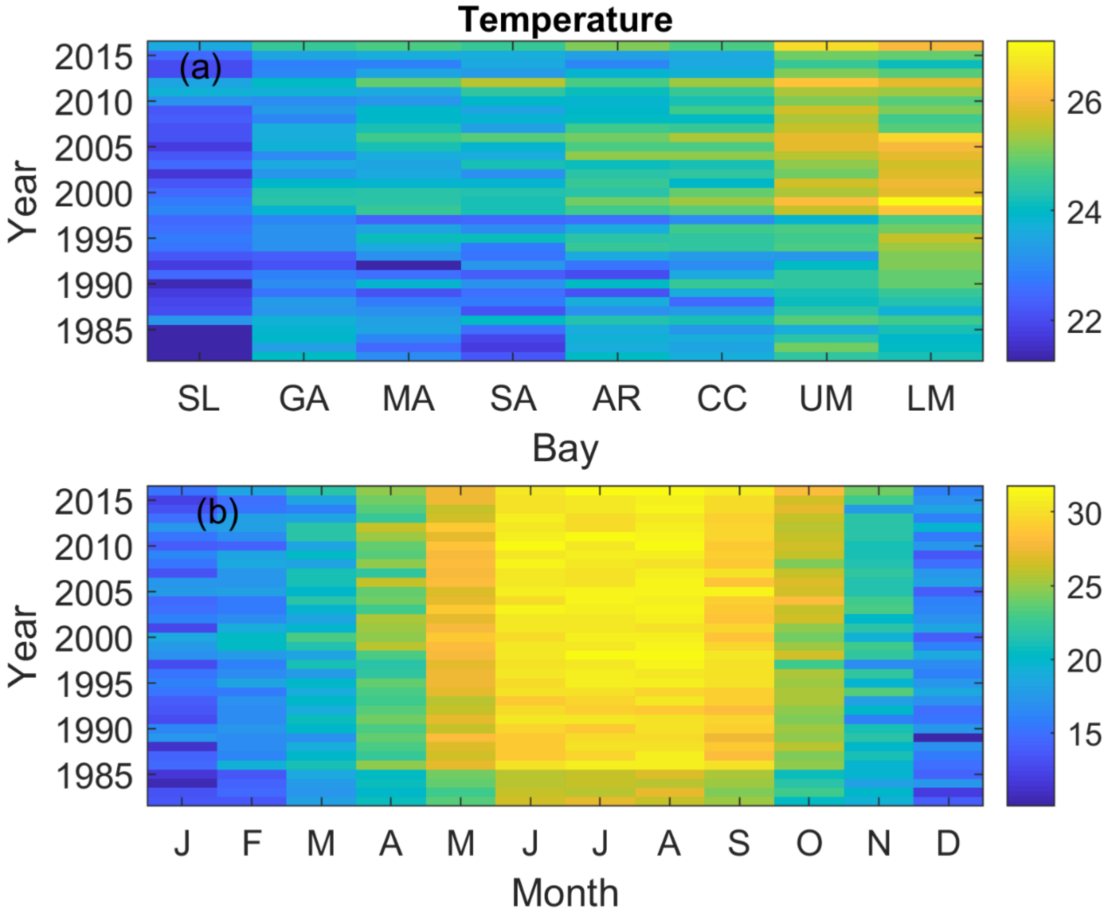

Supplementary Figure 2. Monthly mean water temperature (°C). (a) Averaged over year to show spatial variability, (b) Averaged among locations to show monthly variability. SL: Sabine Lake, GA: Galveston Bay, MA, Matagorda Bay, SA: San Antonio Bay, AR: Aransas Bay, CC: Corpus Christi Bay, UM: Upper Laguna Madre, and LM: Lower Laguna Madre. J-D denote months of a year.

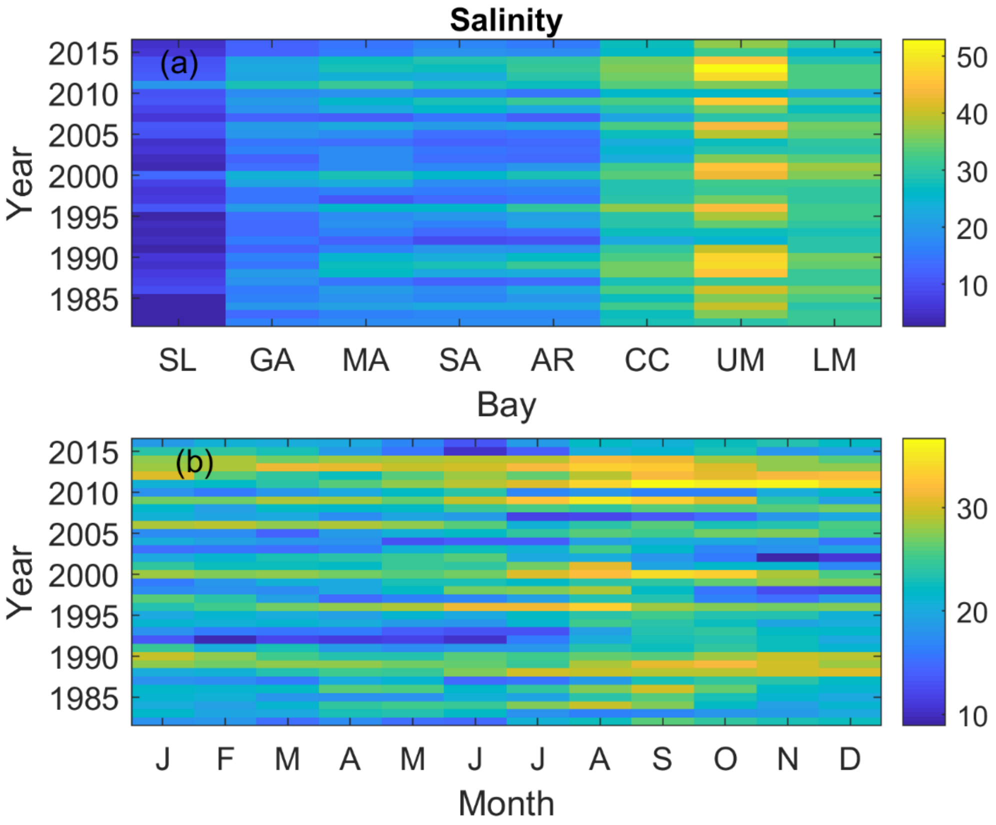

Supplementary Figure 3. Monthly mean salinity (ppt). (a) Averaged over year to show spatial variability, (b) Averaged among locations to show monthly variability. SL: Sabine Lake, GA: Galveston Bay, MA, Matagorda Bay, SA: San Antonio Bay, AR: Aransas Bay, CC: Corpus Christi Bay, UM: Upper Laguna Madre, and LM: Lower Laguna Madre. J-D denote months of a year.

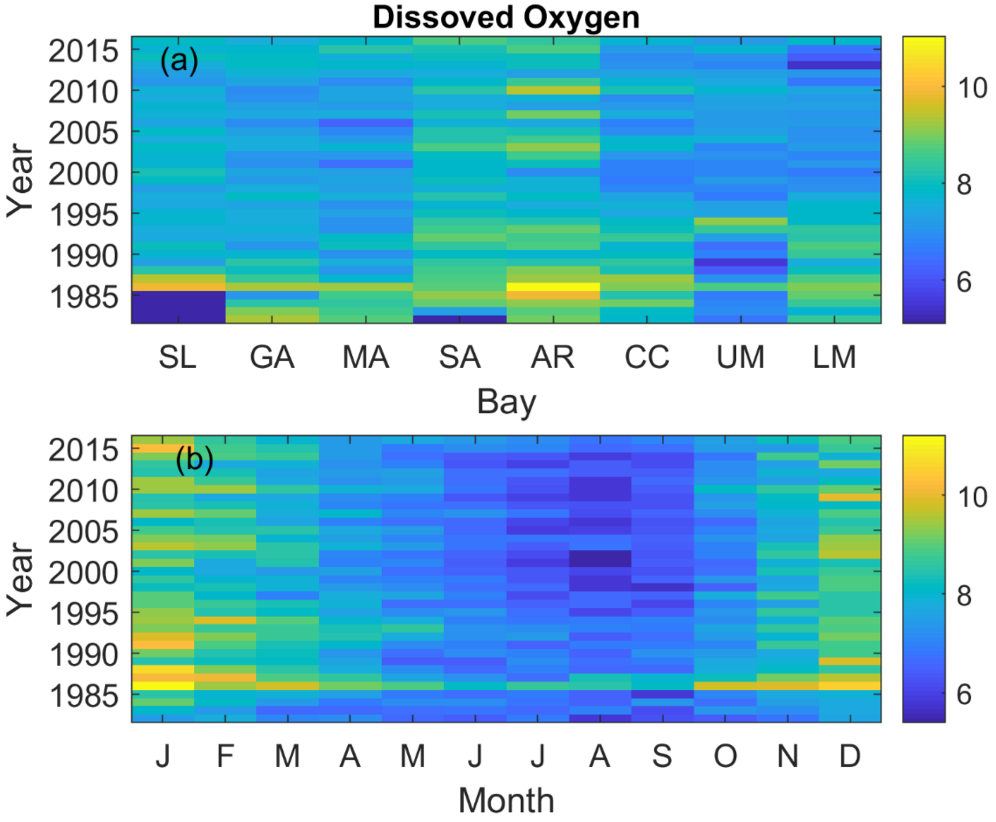

Supplementary Figure 4. Monthly mean dissolved oxygen (ppm). (a) Averaged over year to show spatial variability, (b) Averaged among locations to show monthly variability. SL: Sabine Lake, GA: Galveston Bay, MA, Matagorda Bay, SA: San Antonio Bay, AR: Aransas Bay, CC: Corpus Christi Bay, UM: Upper Laguna Madre, and LM: Lower Laguna Madre. J-D denote months of a year.

Supplementary Table

Supplementary Table 1. Location of bag seine sampling and tide station.

Bay (Bay #)[1]	Tide Station (Station #)[2]
Sabine Lake (1)	Sabine Pass North (8770570)
Galveston Bay (2)	Galveston Pier 21 (8771450)
Matagorda Bay (3)	Rockport (8774770)
San Antonio Bay (4)	Rockport (8774770)
Aransas Bay (5)	Rockport (8774770)
Corpus Christi Bay (6)	Rockport (8774770)
Upper Laguna Madre (7)	Rockport (8774770)
Lower Laguna Madre (8)	Rockport (8774770)

1: Bay # indicate the index used by Texas Parks and Wildlife Department for major bays.

2: The data can be downloaded from https://tidesandcurrents.noaa.gov/. For the present analysis, we used the monthly mean tide level. Station # for tide station indicate station ID used by NOAA Center for Operational Oceanographic Products and Services.
