# Supplementary material for: Climate-related factors cause changes in the diversity of fish and invertebrates in subtropical coast of the Gulf of Mexico
Source: Commun Biol. 2019 Nov 1;2:403. doi: 10.1038/s42003-019-0650-9 (PMC6825143; doi:10.1038/s42003-019-0650-9)
Supplement: Supplementary file 2 — Description of Additional Supplementary Files [file 42003_2019_650_MOESM2_ESM.docx]

# Description of Additional Supplementary Files

## Supplementary Data 1

Sample size for each bay in each month of each year

## Supplementary Data 2

Estimated Shannon diversity index for fish

## Supplementary Data 3

Estimated Shannon diversity index for invertebrates

## Supplementary Data 4

Estimated occupancy probability and related results for each fish species

## Supplementary Data 5

Estimated occupancy probability and related results for each invertebrate species

## Supplementary Data 6

Temporal trends of occupancy probability and associated environmental variables included in the best model for fish

## Supplementary Data 7

Temporal trends of occupancy probability and associated environmental variables included in the best model for invertebrates
