# Supplementary material for: Climate-related factors cause changes in the diversity of fish and invertebrates in subtropical coast of the Gulf of Mexico
Source: Commun Biol. 2019 Nov 1;2:403. doi: 10.1038/s42003-019-0650-9 (PMC6825143; doi:10.1038/s42003-019-0650-9)
Supplement: Supplementary file 10 — Reporting Summary [file 42003_2019_650_MOESM10_ESM.pdf]

nature research

Corresponding author(s): Masami Fujiwara

Last updated by author(s): 2019-03 20

# Reporting Summary

Nature Research wishes to improve the reproducibility of the work that we publish. This form provides structure for consistency and transparency in reporting. For further information on Nature Research policies, see Authors & Referees and the Editorial Policy Checklist.

## Statistics

For all statistical analyses, confirm that the following items are present in the figure legend, table legend, main text, or Methods section.

| n/a | Confirmed | |
|---|---|---|
| ☐ | ☒ | The exact sample size (*n*) for each experimental group/condition, given as a discrete number and unit of measurement |
| ☒ | ☐ | A statement on whether measurements were taken from distinct samples or whether the same sample was measured repeatedly |
| ☐ | ☒ | The statistical test(s) used AND whether they are one- or two-sided<br>*Only common tests should be described solely by name; describe more complex techniques in the Methods section.* |
| ☐ | ☒ | A description of all covariates tested |
| ☐ | ☒ | A description of any assumptions or corrections, such as tests of normality and adjustment for multiple comparisons |
| ☐ | ☒ | A full description of the statistical parameters including central tendency (e.g. means) or other basic estimates (e.g. regression coefficient) AND variation (e.g. standard deviation) or associated estimates of uncertainty (e.g. confidence intervals) |
| ☐ | ☒ | For null hypothesis testing, the test statistic (e.g. $F$, $t$, $r$) with confidence intervals, effect sizes, degrees of freedom and $P$ value noted<br>*Give P values as exact values whenever suitable.* |
| ☒ | ☐ | For Bayesian analysis, information on the choice of priors and Markov chain Monte Carlo settings |
| ☒ | ☐ | For hierarchical and complex designs, identification of the appropriate level for tests and full reporting of outcomes |
| ☒ | ☐ | Estimates of effect sizes (e.g. Cohen's *d*, Pearson's *r*), indicating how they were calculated |

*Our web collection on statistics for biologists contains articles on many of the points above.*

## Software and code

Policy information about availability of computer code

| | |
|---|---|
| Data collection | No software was used. |
| Data analysis | Rarefaction analysis was done with R 3.5.2 with the package iNEXT (Hsieh, et al. 2016). DOI: 10.1111/2041-210X.12613<br>Matlab 9.3 was used for the occupancy model analysis using an optimization routine "fminunc.m". The custom codes for likelihood is available from GitHub (https://github.com) DOI: 10.5281/zenodo.3464538.<br>Matlab 9.3 was used for other statistical analyses. |

For manuscripts utilizing custom algorithms or software that are central to the research but not yet described in published literature, software must be made available to editors/reviewers. We strongly encourage code deposition in a community repository (e.g. GitHub). See the Nature Research guidelines for submitting code & software for further information.

## Data

Policy information about availability of data

All manuscripts must include a data availability statement. This statement should provide the following information, where applicable:

- Accession codes, unique identifiers, or web links for publicly available datasets
- A list of figures that have associated raw data
- A description of any restrictions on data availability

Data used in this analysis are available at BCO-DMO (https://www.bco-dmo.org/dataset/773137). DOI: 10.1575/1912/bco-dmo.773137.1

# Field-specific reporting

Please select the one below that is the best fit for your research. If you are not sure, read the appropriate sections before making your selection.

☐ Life sciences ☐ Behavioural & social sciences ☒ Ecological, evolutionary & environmental sciences

For a reference copy of the document with all sections, see nature.com/documents/nr-reporting-summary-flat.pdf

# Ecological, evolutionary & environmental sciences study design

All studies must disclose on these points even when the disclosure is negative.

| | |
|---|---|
| Study description | The objective of the study was to analyze fish and invertebrate catch data taken from eight major bays along Texas coast from 1982 (from 1986 in one bay) to 2016. The data were collected for Marine Resource Monitoring Program conducted by Texas Parks and Wildlife Department. Therefore, the data were not specifically collected for this project. The scope of the project is to conduct occupancy analysis to determine how the presence/absence of each of 100+ fish and invertebrate species have changed over time, and rarefaction analysis to determine how species diversity has changed over time in the bays. Detailed sampling protocol is described in Marine Resource Monitoring Operations Manual, which is provided with this manuscript. The manual will also be available from BCO-DMO.org along with the raw data. |
| Research sample | Samples were taken by Texas Parks and Wildlife Department as a part of Marine Resource Monitoring Program. Fish and invertebrates were sampled at eight major bays along Texas coast (see the map in the main manuscript). Each deployment of bag seine was treated as one sample, and presence and absence of species was determined for each sample (regardless of the number of individuals observed). There were multiple samples in each month, and data were collected over 35 years (31 years in one location). |
| Sampling strategy | The sample size was pre-determined for the monitoring program (exact sample size is included in Appendix). We did not have any control over the determination of sample size. However, we would like to emphasize that this is one of the largest data set of the kind available for this type of ecological studies. |
| Data collection | The sample was taken with bag seine (18.3 m long and 1.8 m deep with 19 mm stretched nylon mesh in wings and 13 mm stretched mesh in the bag). The bag seine was extended 12.2 m perpendicularly to the shoreline, then pulled parallel to the shoreline over 15.5 m. The offshore end was then retrieved to shore while keeping the onshore end stationary and maintaining the full extent (12.2 m) of the bag seine using a limit line. |
| Timing and spatial scale | The surveys were conducted bi-weekly using bag seine, which was deployed along the shoreline. The coast within each bay was divided into sections, and the sections for sampling were randomly determined each month. |
| Data exclusions | We eliminated species that was only observed less than 30 times (out of 58,604 possible occasions of observations) for occupancy analysis. This is because we did not expect to be able to estimate parameters in an occupancy model for those species. We further eliminated species for which parameters were not estimable even though they may be observed more than 30 times. |
| Reproducibility | The very clear sampling protocol has been developed by Texas Park and Wildlife Department (see the operation manual), and sample size is large for ecological study. Therefore, although exactly same data are not expected to be reproducible, data should be representative of the sampled popualtions. |
| Randomization | The sampling employed nested random sample (i.e. random selection within a bay). |
| Blinding | Blinding (as a term used in clinical trial) was not used in this study. |

Did the study involve field work? ☐ Yes ☒ No

# Reporting for specific materials, systems and methods

We require information from authors about some types of materials, experimental systems and methods used in many studies. Here, indicate whether each material, system or method listed is relevant to your study. If you are not sure if a list item applies to your research, read the appropriate section before selecting a response.

## Materials & experimental systems

| n/a | Involved in the study | |
|---|---|---|
| ☒ | ☐ | Antibodies |
| ☒ | ☐ | Eukaryotic cell lines |
| ☒ | ☐ | Palaeontology |
| ☒ | ☐ | Animals and other organisms |
| ☒ | ☐ | Human research participants |
| ☒ | ☐ | Clinical data |

## Methods

| n/a | Involved in the study | |
|---|---|---|
| ☒ | ☐ | ChIP-seq |
| ☒ | ☐ | Flow cytometry |
| ☒ | ☐ | MRI-based neuroimaging |
